# Assessing quantity combination and dissociation in giraffes

**DOI:** 10.1038/s41598-026-54126-7

**Published:** 2026-06-26

**Authors:** Iker Loidi, Álvaro L. Caicoya, Federica Amici, Pilar Padilla-Solé, Jordi Galbany

**Affiliations:** 1 Department of Clinical Psychology and Psychobiology, Faculty of Psychology, University of Barcelona, Barcelona, Spain; 2 Institute of Neurosciences, University of Barcelona, Barcelona, Spain; 3 Research Institute for Farm Animal Biology (FBN), Dummerstorf, Germany; 4 Research Group Human Biology and Primate Cognition, Institute of Biology, University of Leipzig, Leipzig, Germany; 5 Department of Comparative Cultural Psychology, Max Planck Institute for Evolutionary Anthropology, Leipzig, Germany; 6 Barcelona Zoo, Barcelona, Spain

**Keywords:** Arithmetic, Ungulates, Numerical cognition, Quantity discrimination, Numerosity, Object permanence, Ecology, Ecology, Neuroscience, Psychology, Psychology, Zoology

## Abstract

Several species have demonstrated the ability to mentally manipulate quantities in tasks requiring their combination and dissociation, reflecting perceptual memory–based mechanisms that may be the foundation of proto-arithmetic and in some cases more advanced arithmetic abilities. Yet, ungulates remain underrepresented in this line of research. Here, we present the first investigation in giraffes (*Giraffa camelopardalis*) assessing their ability to combine and dissociate quantities. We tested four captive giraffes in three experimental and two controls tasks. In the experimental tasks, the animals could only see the initial quantities and the items moved, but not the final amounts. Results revealed that giraffes performed above chance in the Combination task (where food was combined to one of two covered quantities). However, they performed at chance level in both the Dissociation task (where food was removed from one of two covered quantities) and the Subsequent Events task (where food was transferred from one of the two quantities to the other one). Further analyses suggested that while two subjects might have relied on simpler strategies (e.g., choosing or avoiding the dish manipulated by the experimenter), the other two succeeded even when this strategy could not be used, suggesting the potential use of more complex mental computations.

**Supplementary Information:**

The online version contains supplementary material available at 10.1038/s41598-026-54126-7.

## Introduction

To uncover the evolutionary origins of human numerical cognition, researchers have studied different numerical skills across a wide range of species^1–3^. One of the most fundamental numerical skills is the ability to estimate differences between sets, either by relying on overall quantity (including continuous variables such as mass, volume or area occupied) or on discrete numerosity (evaluating the exact number of items in the set)^4^. When relying on overall quantity, animals demonstrate quantity discrimination, an ability that has been found in several taxa, including invertebrates^5–7^, fish^8–11^, amphibians^12,13^, reptiles^14–18^, birds^19–21^ and mammals^22–30^. In contrast, when relying solely on the number of items, because continuous variables are controlled for, animals show numerical discrimination, which has been reported in invertebrates^31–33^, amphibians^34,35^, fish^36^, birds^37–42^, and primates^43–45^. These findings have challenged early theories of numerical skills, which suggested that the ability to process quantitative or numerical information required language and symbolic representations, abilities once thought to be uniquely human^46^.

Numerical skills rely on two different non-symbolic cognitive systems that can operate in parallel^47^. The analogue magnitude system (AMS) appears to be a core system available to most animals^1^. It allows approximate numerical representations and is ratio-dependent in accordance with Weber’s law (i.e., discrimination accuracy depends on the ratio between quantities, with closer ratios being harder to distinguish)^48^. In contrast, the object file system (OFS) represents each perceived entity individually by encoding them as object files, and it typically supports the representation of small numerosities (up to four)^49,50^. The OFS has been identified in fewer non-human species, where it functions alongside the AMS^10,13,18,20,32,35,40^.

Several studies have shown that animals have other numerical skills beyond simple quantity or numerical discrimination^51–63^. Arithmetic reasoning, for instance, involves both forming internal numerical representations and mentally manipulating them through operations such as additions (i.e., the mental combination of two or more quantitative representations to obtain a new total) or subtractions (i.e., modifying an existing representation by removing a smaller quantity to obtain the remainder), making it more cognitively demanding than simpler quantity or numerical discrimination tasks^63,64^. In recent years, several studies have demonstrated addition abilities using symbolic paradigms in which animals are trained to associate symbols with exact numerosities. Studies with the chimpanzee (*Pan troglodytes*) Sheba and the African grey parrot (*Psittacus erithacus*) Alex have shown that these species can use Arabic numerals to perform additions, reaching sums of up to four and eight, respectively^65,66^. Moreover, corvids (*Corvus cornix*)^67^, pigeons (*Columba livia*)^68^, capuchin monkeys (*Cebus apella*)^69^ and squirrel monkeys (*Saimiri sciureus*)^70^ have also demonstrated addition abilities using different symbolic systems, whereas honeybees (*Apis mellifera*) have been shown to learn to use color cues to add a single element to small arrays^71^.

Most studies conducted to date, however, have relied on on non-symbolic paradigms that require no extensive training^63^. These approaches allow testing whether animals can track quantity changes after combining items in a set (i.e., mentally integrating two or more separate quantities into a single unified representation) or dissociating them (i.e., mentally separating a portion of a quantity from the whole). While such abilities may appear proto-arithmetical when subjects rely solely on number^72–79^, they do not necessarily imply true number-based processing. Depending on the set-up, animals may instead rely on continuous variables to encode, compare and manipulate amounts mentally^80^. This sensitivity to changes in quantity may rely on memory-based perceptual mechanisms that allow retaining and comparing previous quantities, guiding decisions about current or future amounts^80^. As these tasks require animals to maintain mental representations of quantities that are manipulated out of view, they are more complex than simple discrimination tasks and may underlie more advanced proto-arithmetic or symbol-based arithmetic abilities^80^.

One approach that has been used to test combinations and dissociations is the violation-of-expectation paradigm, in which individuals are considered to solve the task if they show signs of surprise, such as longer looking time or behavioral hesitation, when presented with unexpected results^81^. Under this paradigm several species could mentally compute combinations of up to 3 items (domestic dogs: *Canis familiaris*^82^; various species of lemurs: *Eulemur fulvus*,* E. mongoz*,* Lemur catta* and *Varecia rubra*^75^; and cotton-top tamarins: *Saguinus Oedipus*^72^), or even up to 8 items (rhesus macaques: *Macaca mulatta*^74^). However, this methodology has important limitations, as it relies on indirect behavioural measures that may reflect perceptual or attentional responses rather than true numerical cognition^83–85^. Therefore, alternative paradigms relying on direct behavioural choices have been developed. In some of these tasks, items are sequentially hidden within opaque containers, forcing the individual to mentally compute the final, unseen combination to make a correct selection. These approaches suggest a phylogenetic divide, with primates successfully combining multiple items under these conditions (rhesus macaques^77^, chimpanzees^86–88^), and non-primate species showing more limited performance (dogs: *Canis lupus familiaris*^89^; zebrafish: *Danio rerio*^90^; and horses: *Equus ferus caballus*^73,89,90^). Using a similar paradigm in which the initial quantities were different from 0 and just one of the options was manipulated, orangutans (*Pongo pygmaeus*) solved combinations with up to six items^80^. Finally, when presented with two options, each containing two sets of different quantities to be mentally combined, chimpanzees managed to combine up to six items^91^, while rhesus macaques were able to track the accumulation up to 16 items, with total sets reaching up to 17 (e.g., 16 + 1)^52^.

Although some species can perform combinations, research on the ability to perform dissociations is much scanter. After intensive training, pigeons could successfully peck the correct key even in dissociations such as 12 − 6^92^. Under a less complex, non-training-based paradigm, orangutans could also solve dissociations with up to six items, although their performance was lower in dissociation than in combination tasks and appeared to partially rely on simpler cognitive strategies^80^. Remarkably, with a similar set-up, newborn chicks (*Gallus gallus*) were able to perform dissociations of up to three items from sets of five^78^. However, under the violation-of-expectation paradigm, rhesus monkeys showed no signs of surprise during a dissociation task, regardless of the outcome, suggesting they lacked clear expectations following the removal of items^93^. When tested with a different paradigm, semi-wild vervet monkeys appeared to solve dissociations in which items were removed simultaneously, but not sequentially^64^. Similarly, when removing items one by one in a choice task, chimpanzees achieved significant results only when a single item was removed, but not more than one^87^. Overall, these findings suggest that dissociations may be more cognitively demanding than combinations.

Finally, few studies have assessed whether animals can sequentially perform dissociations and combinations, by assessing if they can combine to one option what has previously been removed from another. This ability requires not only being able to track accumulated or removed items, but also successfully monitoring their movement between quantities, while keeping initial quantities in mind, which places high demands on working memory^78^. When tested with this set-up, chicks could sequentially remove and combine up to three items, whereas rhesus macaques were only capable of tracking the removal and subsequent combination of a single reward^78,94^.

To the best of our knowledge, none of these paradigms used to test abilities based on quantity manipulation has yet been applied to the study of non-domestic ungulates. In particular, whereas several non-domesticated ungulate species can discriminate quantities^28,95^, their ability to mentally manipulate them has not been explored. Giraffes (*Giraffa camelopardalis*), for instance, can discriminate quantities using approximate number representations (i.e., using the AMS) and show basic statistical inference abilities, choosing the option more likely to provide favorite food^95,96^. As browsing herbivores, giraffes feed on dispersed food sources and show a dietary breadth comparable to that of chimpanzees^97^. Additionally, they form social groups characterized by high levels of fission-fusion dynamics and live in open ecosystems with abundant predators^98^. This ecological and social complexity might be linked to the enhancement of specific cognitive skills, including numerical skills, which could be adaptive to select more abundant food sources, avoid areas with higher predator density and more effectively monitor changes in their frequently changing social groups^3,24,99,100^.

In this study, we aimed to test whether giraffes can mentally combine or dissociate discrete quantities in a spontaneous choice task. To this end, we adapted a version of a choice-task originally developed by Call^80^ to study orangutans. Using this methodology, we implemented two different tasks: the Combination (COMB) and the Dissociation (DISS) tasks. Moreover, we included a third task, the Subsequent Events (SUBE), adapted from Rugani and colleagues^78^, to assess whether giraffes can also process sequential events (i.e., by first removing items from one option, and then combining them to the other option). Considering the skills previously demonstrated by these animals in this domain, and literature in other species, we predicted that giraffes would succeed in the COMB task, by preferentially choosing the container with the larger final quantity (Prediction 1), but not in the DISS task (Prediction 2) and in the SUBE task (Prediction 3), as both appear to be more cognitively demanding than the COMB task.

We also examined whether performance could be explained by the use of simpler strategies, including the DMO strategy (*Dish Manipulated by the Observer*), the ILQ strategy (*Initial Largest Quantity*) and the SLQ strategy (*Single Largest Quantity*)^80^. The DMO occurs when the animal preferentially selects the container manipulated by the experimenter during the COMB task and avoids it during the DISS task. The ILQ involves choosing the container that initially held the largest number of items, regardless of subsequent manipulations. The SLQ strategy involves selecting the container that, by the end of the trial, held the largest quantity among the ones the animal was presented at the beginning of the trial (i.e., the two initial quantities in the two yellow containers, and the amount initially contained in the green container then moved to one of the yellow containers; see Methods for a more detailed explanation).

## Results

### Giraffes perform differently across tasks

**Fig. 1:**
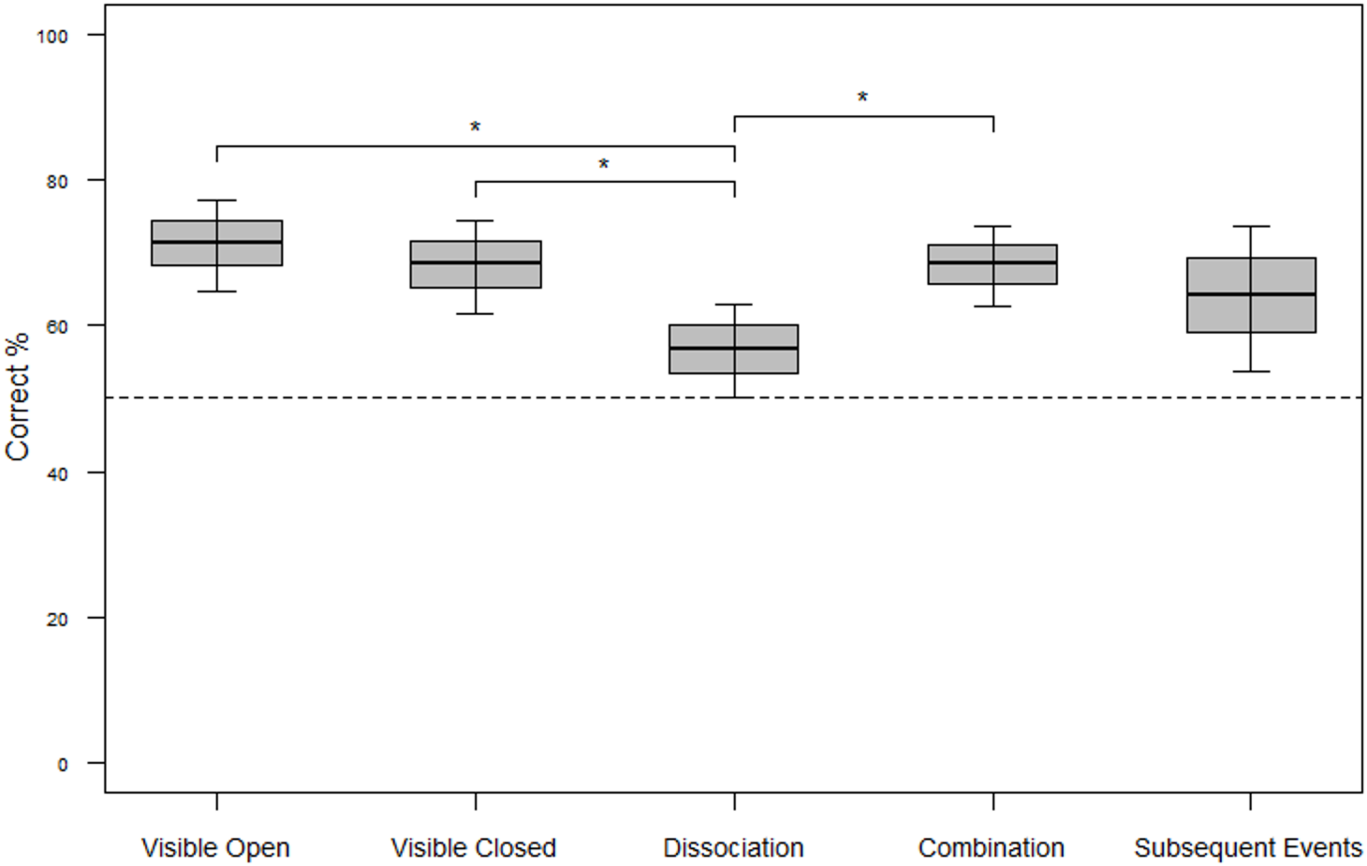
Percentage of correct trials by task. The thick lines within the box plots indicate the mean percentages for each task, as estimated by Model 1. The edges of the boxes represent the estimated means ± standard errors, while the whiskers denote the 95% confidence intervals. In Model 1, the probability of success was significantly higher for the Combination, Visual Open and Visual Closed tasks compared to the Dissociation task.

In Model 1, we assessed how individuals performed in the different tasks, by comparing performance across tasks, while controlling for the ratio between sets, possible learning effects and side biases (see Methods). The full-null model comparison was significant (Table 1), with task having a significant effect on individuals’ performance (*p* = 0.017). Post hoc comparisons revealed that performance was lower in the DISS task, as compared to the COMB task (*odds ratio* = 1.65, *z* = 2.63, *p =* 0.009) and to both control tasks in which giraffes had to discriminate quantities without manipulations, both when the quantities were visible (Visible Open [VO]: *odds ratio* = 0.53, *z* = −3.07, *p* = 0.002) or not (Visible Closed [VC]: *odds ratio* = 0.60, *z* = −2.44, *p* = 0.015) at the time of choice (Fig. 1).

**Table 1:** Summary of the two models run. We report the estimates, standard errors (SE), confidence intervals (CIs), likelihood ratio tests (LRT), degrees of freedom (*df*), and *p*-values for each predictor and control (in italics). Significant predictors are marked with an asterisk in bold, tendencies are marked just with an asterisk in parenthesis, and reference categories for categorical variables are indicated in parentheses.

Models	Estimate	SE	2.5% - 97.5% CI	LRT	df	*p*
Model 1: Model with all tasks (GLMM: *χ2* = 12.09, *df* = 4, *p* = 0.017*)						
Intercept	0.80	0.23	0.35 to 1.25	—	—	—
Task (Visual Open)	0.14	0.20	−0.25 to 0.53	12.09	4	0.017*
Task (Visual Closed)	0.00	0.20	−0.38 to 0.39			
Task (Dissociation)	−0.50	0.20	−0.88 to −0.13			
Task (Subsequent Events)	−0.20	0.25	−0.69 to 0.31			
*Initial Ratio*	−0.32	0.28	−0.87 to 0.23	1.32	1	0.250
*Session*	0.07	0.06	−0.04 to 0.18	1.40	1	0.237
*Correct Position (Right)*	−0.10	0.13	−0.36 to 0.15	0.64	1	0.423
Model 2: Model without control tasks (GLMM: *χ2* = 12.82, *df* = 5, *p* = 0.025*)						
Intercept	0.32	0.67	−0.98 to 1.63	—	—	—
Task (Dissociation)	−0.60	0.25	−1.09 to −0.11	5.85	2	0.054^(*)^
Task (Subsequent Events)	−0.14	0.28	−0.69 to 0.41			
Number of items moved	0.01	0.12	−0.24 to 0.25	0.00	1	0.954
SLQ	0.30	0.23	−0.17 to 0.77	1.59	1	0.207
DMO	0.53	0.28	−0.02 to 1.08	3.68	1	0.055^(*)^
*Initial Ratio*	−0.35	0.50	−1.34 to 0.64	0.49	1	0.486
*Final Ratio*	0.05	0.48	−0.88 to 0.99	0.01	1	0.912
*Session*	0.02	0.07	−0.12 to 0.16	0.11	1	0.745
*Correct Position (Right)*	−0.02	0.16	−0.34 to 0.30	0.02	1	0.895

On average (mean ± SE), subjects made the correct choice in 68 ± 3% of the trials in the COMB task, in 57 ± 3% of the trial in the DISS task, and in 64 ± 5% of the trials in the SUBE task. Binomial tests of proportions further showed that all subjects performed significantly above chance in the COMB task (Table 2), while their performance in the DISS and SUBE tasks decreased at chance level (Table 2). With the exception of Nuru in VC, all subjects performed above chance in both control tasks (Table 2). The individual percentage of correct responses for each task and condition is available in the Supplementary Tables.

**Table 2:** Individual performance as a function of task and the use of DMO strategy. For each individual, we report the relative number of correct responses (Correct), the mean probability of success (Probability), confidence intervals (CIs), and *p*-values of the binomial proportion tests. Significant results are marked with an asterisk in bold.

Individual	Task	Strategy	Correct	Probability	2.5% - 97.5% CI	*p*
Nuru	Visual Open		39/52	0.75	0.61 to 0.86	<0.001*
	Visual Closed		32/52	0.62	0.47 to 0.75	0.126
	Combination		53/72	0.74	0.62 to 0.83	<0.001*
	Dissociation		39/72	0.54	0.42 to 0.66	0.556
	Subsequent Events		16/24	0.67	0.45 to 0.84	0.152
		DMO	74/116	0.64	0.54 to 0.73	0.004*
		No DMO	34/52	0.65	0.51 to 0.78	0.037*
Nakuru	Visual Open		37/52	0.71	0.57 to 0.83	0.003*
	Visual Closed		34/52	0.65	0.51 to 0.78	0.036*
	Combination		49/72	0.68	0.56 to 0.80	0.003*
	Dissociation		38/72	0.53	0.41 to 0.65	0.724
	Subsequent Events		17/24	0.71	0.50 to 0.87	0.064
		DMO	75/116	0.65	0.55 to 0.73	0.002*
		No DMO	29/52	0.56	0.41 to 0.70	0.489
Njano	Visual Open		36/52	0.69	0.55 to 0.81	0.008*
	Visual Closed		35/52	0.67	0.53 to 0.80	0.018*
	Combination		48/72	0.67	0.55 to 0.77	0.006*
	Dissociation		42/72	0.58	0.46 to 0.70	0.195
	Subsequent Events		16/24	0.67	0.45 to 0.84	0.152
		DMO	71/116	0.61	0.52 to 0.70	0.020*
		No DMO	35/52	0.67	0.53 to 0.80	0.018*
Yalinga	Visual Open		37/52	0.71	0.57 to 0.83	0.003*
	Visual Closed		42/52	0.81	0.67 to 0.90	<0.001*
	Combination		48/72	0.67	0.55 to 0.78	0.006*
	Dissociation		39/72	0.54	0.42 to 0.66	0.556
	Subsequent Events		14/24	0.58	0.37 to 0.78	0.541
		DMO	72/116	0.62	0.53 to 0.71	0.012*
		No DMO	29/52	0.56	0.41 to 0.70	0.489

### Individual differences in the use of simpler strategies

In Model 2, we tested which factors affected performance in the COMB, DISS and SUBE tasks, and whether subjects relied on simpler strategies to solve the tasks. In particular, Model 2 tested whether performance was affected by the number of items moved and by the potential use of ILQ, SLQ and DMO strategies, while controlling for the ratio between sets, possible learning effects and side biases. In this model, multicollinearity analysis revealed a high correlation between ILQ and SLQ (see Methods), as most trials solvable by the ILQ strategy were also solvable using SLQ. As a result, ILQ was removed from the model and the model re-run. This model revealed a significant difference from the null model (Table 1), with both task (*p* = 0.054) and DMO strategy (*p* = 0.055) having marginally significant effects on individuals’ general performance. Post hoc comparisons for task confirmed that individuals performed better in the COMB than in the DISS task (*odds ratio* = 1.82, *z* = 2.40, *p =* 0.016). Moreover, using the DMO strategy marginally increased individuals’ general performance. In contrast, neither the use of the SLQ strategy (*p* = 0.207) nor the number of items being moved (*p* = 0.954) had a significant effect on performance.

Binomial tests of proportions further revealed that all subjects performed above chance in the trials where the DMO strategy was effective for solving the trials (Table 2). However, two subjects (i.e., Nuru and Njano) also performed above chance in the trials where using the DMO strategy would have led to failure (Table 2).

## Discussion

Using a non-symbolic choice paradigm, we evaluated the ability of four captive giraffes to mentally represent and manipulate quantities. Giraffes were able to locate the largest quantity after mentally combining quantities in the Combination task (COMB), but they failed to do so when items were removed during the Dissociation task (DISS), or when items removed from one option were combined to the other one in the Subsequent Event task (SUBE). All subjects performed above chance level in the Visible Open (VO) and in the Visible Close (VC) control tasks, in which initial quantities were not manipulated, except for Nuru in the VC task. We further detected an overall tendency for individuals to rely on the DMO (*Dish Manipulated by the Observer*) strategy when attempting to solve the experimental tasks, although two individuals reliably selected the larger option also when the DMO strategy could not be used.

Overall, subjects were able to solve the control conditions, showing that they could reliably discriminate the initial quantities even when, at the time of choice, the initial amounts were not visible (i.e., VC). The only exception was Nuru, who performed below chance in the VC task, despite succeeding in the more cognitively demanding COMB task. This distinct pattern suggests that Nuru’s engagement might have been context-dependent: while the lower complexity of the VC task may have failed to sustain sufficient attention, the manipulation events in the COMB task potentially triggered the higher engagement required to succeed. Aside from this instance, results suggest that failure to select the larger option in the experimental tasks (COMB, DISS, SUBE) could only depend on the inability of giraffes to understand the manipulation of the initial quantities, rather than to a lack of (i) motivation, (ii) preference for the larger quantity and/or (iii) ability to select the larger of two non-visible quantities.

In line with Prediction 1, giraffes were able to correctly solve the COMB task, performing significantly above than chance, and comparably to the VC and VO control conditions, where there was no manipulation. This suggests that giraffes were able to mentally track combinations or cumulative manipulations of the initial quantities and correctly compare the outcomes, possibly using a simple perceptual memory–based mechanism that enabled them to follow these combinations and make accurate quantitative decisions, as proposed by Call (2000) in orangutans^80^. In this task, giraffes managed to mentally accumulate up to three elements to previously seen quantities, reaching combinations of up to five, and thus performing similarly to some primates under similar experimental conditions^77,80^. Their performance was also better than species tested using the controversial violation-of-expectations paradigm, such as dogs^82^, lemurs^75^, and tamarins^72^, and species tested with choice-based tasks, such as zebrafish^90^ and horses^73^, but lower compared to that of chimpanzees^86–88,91^ or pigeons^68^. However, comparisons between studies using different approaches should be interpreted with caution, and future studies should ideally apply the same procedures across species to reliably detect inter-specific differences. Our results also show that giraffes could combine items sequentially, a skill that requires working memory and has been observed in other mammals^72–75,77,86–89^, although in our case the cognitive demands were somewhat lower, as the maximum number of items accumulated was three.

We found no evidence that giraffes could solve dissociations (in line with Prediction 2). No subject performed above chance in this task, and performance in the DISS task was significantly lower than that observed in the COMB, VC and VO tasks. These results are consistent with previous studies showing that several species can solve combination but not dissociation tasks, especially when having to track the dissociation of more than one item or do so sequentially, as observed in chimpanzees, vervet monkeys, and macaques^64,87,93^. Orangutans and pigeons achieved better results; however, orangutans’ performance seemed to rely on simpler strategies rather than on true mental quantitative processing^80^, while pigeons were evaluated only after extensive training, which makes direct comparisons to our study difficult^92^. Taken together, our results align with literature and support the idea that dissociation tasks are cognitively more demanding than combination tasks. In line with this, neurological research in humans demonstrates that subtractions require longer response times and engage brain regions linked to controlled rather than automatic processing, such as the right prefrontal cortex and parietal areas^101^. Developmentally, subtraction skills emerge later than addition in children, who often rely on “subtraction by addition” strategies (e.g., solving 10 − 3 by asking how much must be added to 3 to reach 10)^102,103^.

In line with Prediction 3, giraffes also failed in the SUBE task, which required the capacity to track the removal of items from one option and then their combination to the other option. This ability requires accurately tracking not only changes in quantity, but also the movement of individual items from one invisible quantity to the other, while remembering those initial quantities^78^. This involves a level of cognitive complexity and a working memory load that appears to have limits even in humans, as children tested under similar experimental conditions showed difficulties to pass this task^104^. Notably, newborn chicks achieved significant success in this test^78^. According to the authors, this might depend on the fact that chicks usually have 8 to 10 siblings, and thus might have evolved quantitative skills to specifically monitor group size within a small area around the mother. However, it is also possible that, having been imprinted at birth with the visual stimuli later used in the experiments, their attention and motivation to track the items during the task increased^78^. In line with this, older individuals of the same species, who had not undergone imprinting, struggled at solving similar tasks^40^. Moreover, our results might also depend on the fact that, to avoid learning effects, we only administered a limited number of trials in this task. Indeed, post-hoc tests showed a significant difference between the COMB and DISS tasks, but not between the SUBE and the other tasks, with individual performances being very similar in the COMB and SUBE tasks for most giraffes (Nuru: 73.61% in COMB and 66.67% in SUBE; Njano: 66.67% in both COMB and SUBE; Nakuru: 68.06% in COMB and 70.83% in SUBE). In particular, whereas binomial tests for 72 trials (COMB, DISS) reach significance when at least 62.5% of the trials are correct, at least 75.0% of correct choices are needed to reach significance with only 24 trials, as in SUBE. Thus, the lack of significance in this condition could also depend on the low number of observations.

In terms of potential reliance on simpler strategies, only one strategy (DMO) showed an almost significant effect on the general probability of correctly solving the tasks: individuals tended to choose the option manipulated by the experimenter in the COMB task and avoid it in the DISS task. In tasks involving quantitative manipulations, other species have shown patterns consistent with the use of simpler strategies, such as those analysed in this study. For example, orangutans exhibited behaviours consistent with DMO and SLQ strategies in both combination and dissociation tasks^80^, and one chimpanzee showed performance consistent with the ILQ strategy in a task involving the combination of sets differing in numerosity^86^. In our case, it was not possible to directly evaluate the ILQ strategy, as it was highly correlated with SLQ; but as SLQ showed no significant effect, we can confidently state that neither of these two strategies explained the response of giraffes in our study.

While relying on the DMO tendentially improved overall performance, there were differences between individuals: Nakuru and Yalinga performed above chance only in trials that could be solved using the DMO, while Nuru and Njano were successful even when this strategy could not be used to select the larger final reward. Therefore, two individuals solved the tasks without solely relying on these simpler strategies. Moreover, these results reflect considerable individual variation, in line with other studies on orangutans, chimpanzees, vervet monkeys and dogs^64,80,86,89,91^, and suggest that individual differences, including developmental experiences, may contribute to explain variation in skills involving the manipulation of quantities. Incidentally, it should be noted that Nuru has excelled also in previous numerical tasks^95,96^.

The number of pieces moved during the manipulation had no significant effect on general performance, and we also found no significant side biases or learning effects. The lack of learning effects is easily explained by the low number of trials we administered (i.e., 4 trials for each specific task and quantity combination, which were distributed across different sessions). Moreover, higher initial ratios between items had a negative effect on performance, despite not significant, in line with Weber’s law and with previous evidence showing that these animals can discriminate quantities by means of approximate quantitative representations^95^, confirming the involvement of the AMS during basic combination and dissociation tasks.

Our study has several important limitations. First, the number of participants was very low, which limits the strength of our conclusions. Future studies should aim to increase the number of individuals or to apply the same methodology to other species to assess the results from a comparative perspective. Second, the present study examines the use of overall quantity as a cue to understand processes involving the combination or dissociation of quantities, but it does not determine which specific aspect or dimension of quantity giraffes attend to, as our set-up did not disentangle between number of items, mass, surface area and volume. We know that giraffes can discriminate quantities based on number, but mass also influences their performance: giraffes could choose the larger option when differences involved mass rather than number (e.g., 1 vs. 1 items of different mass), but performance declined when mass and number provided conflicting information, with better accuracy when number was the more reliable cue^95^. These results indicate that giraffes use both mass and number to discriminate quantities and highlight the need for further studies specifically assessing reliance on different aspects of quantity during tasks that involve the manipulation of quantities, in order to determine whether the combination abilities observed in this species can support more complex proto-arithmetic or perhaps even arithmetic skills. Additionally, the manipulation of choice options required the experimenter to manually move the items and subsequently raise the containers to allow the subject to make a selection. Despite the experimenter’s experience and strict control of visual, postural and facial cues (see Methods), this manual procedure may have influenced selection through subtle, unintentional signals provided to the animals. Future studies should thus prioritize automated and blind methods. Finally, although we overall conducted more than a thousand trials, in some tasks the number of trials might have been too low to detect significant trends in the presence of moderate effects (i.e., SUBE). Future studies with larger samples would be able to solve this issue.

In conclusion, our findings show that at least two giraffes were able to reliably succeed in tasks involving the simple combination of items, without relying on simpler strategies, and suggest that giraffes may be capable of mentally tracking quantity manipulations, likely using perceptual memory–based mechanisms, to identify the location of the larger food quantity. However, giraffes failed the more cognitively demanding DISS and SUBE tasks. Our results extend previous research showing that giraffes can discriminate quantities and make statistical inferences^95,96^, and indicate that their quantitative skills may be broader than commonly assumed. These skills might have evolved in response to the socio-ecological pressures that giraffes face in the wild, such as the need to track conspecifics and their relationships in groups with high fission–fusion levels^3,24,99^ or having to deal with a varied diet that requires knowing and remembering the location of different food sources^100^. This study contributes to the growing evidence that complex quantitative skills are not exclusive to primates but may also be present in relatively smaller-brained animals like giraffes. Understanding the distribution of these skills in species other than primates will allow us to shed light on the socio-ecological pressures that are linked to their emergence, and to assess whether the same evolutionary forces are at work across different taxa.

## Methods

### Ethics

This study was approved by the Barcelona Zoo before the onset of data collection. The study strictly adhered to the legal and ethical regulations of the country in which it was conducted (Spain), as the Barcelona Zoo is recognized as an authorized centre for animal research in accordance with the Royal Decree 53/2013^105^ and it also complies with the ARRIVE guidelines^106^. The Comité de Ética y Bienestar Animal (CEBA; Animal Ethics and Welfare Committee) from the Barcelona Zoo and Fundació Barcelona Zoo, including the General Curator, the Mammals Curator, and the keepers working with the species, assessed the experimental procedures and classified the study as a form of enrichment for the giraffes, thus no additional permits were necessary. During the study, all subjects received a daily diet consisting of hay available ad libitum, along with restricted daily portions of pellets and vegetables, and they were never deprived of food or water. During the testing phase, individuals remained in their social group and were only tested when they approached the experimenter voluntarily, being always completely free to leave and interrupt testing.

### Subjects

We conducted our study with four giraffes (*Giraffa camelopardalis*), two males (Nakuru: 8 years old; Njano: 7 years old) and two females (Nuru: 15 years old; Yalinga: 21 years old), housed at the Barcelona Zoo. The subjects were born in captivity and belong to a lineage that has lived in zoological parks for at least two other generations. The giraffes were exposed to humans during routine activities, such as feeding, cleaning, and transfers between different sections of their enclosures. The experimenter (IL) however, only interacted with the subjects during the experiments, which began after a brief habituation phase designed to familiarize the subjects with the study materials. All study subjects had been previously tested in other cognitive studies^107,108^, including two on numerical cognition^95,96^.

### Experimental procedure

We administered three experimental tasks, in which we showed two different food quantities to the subjects. After being covered and manipulated, we presented the quantities to the subjects, which could select the option providing the larger amount, by mentally computing the outcome of the manipulation.

**Fig. 2:**
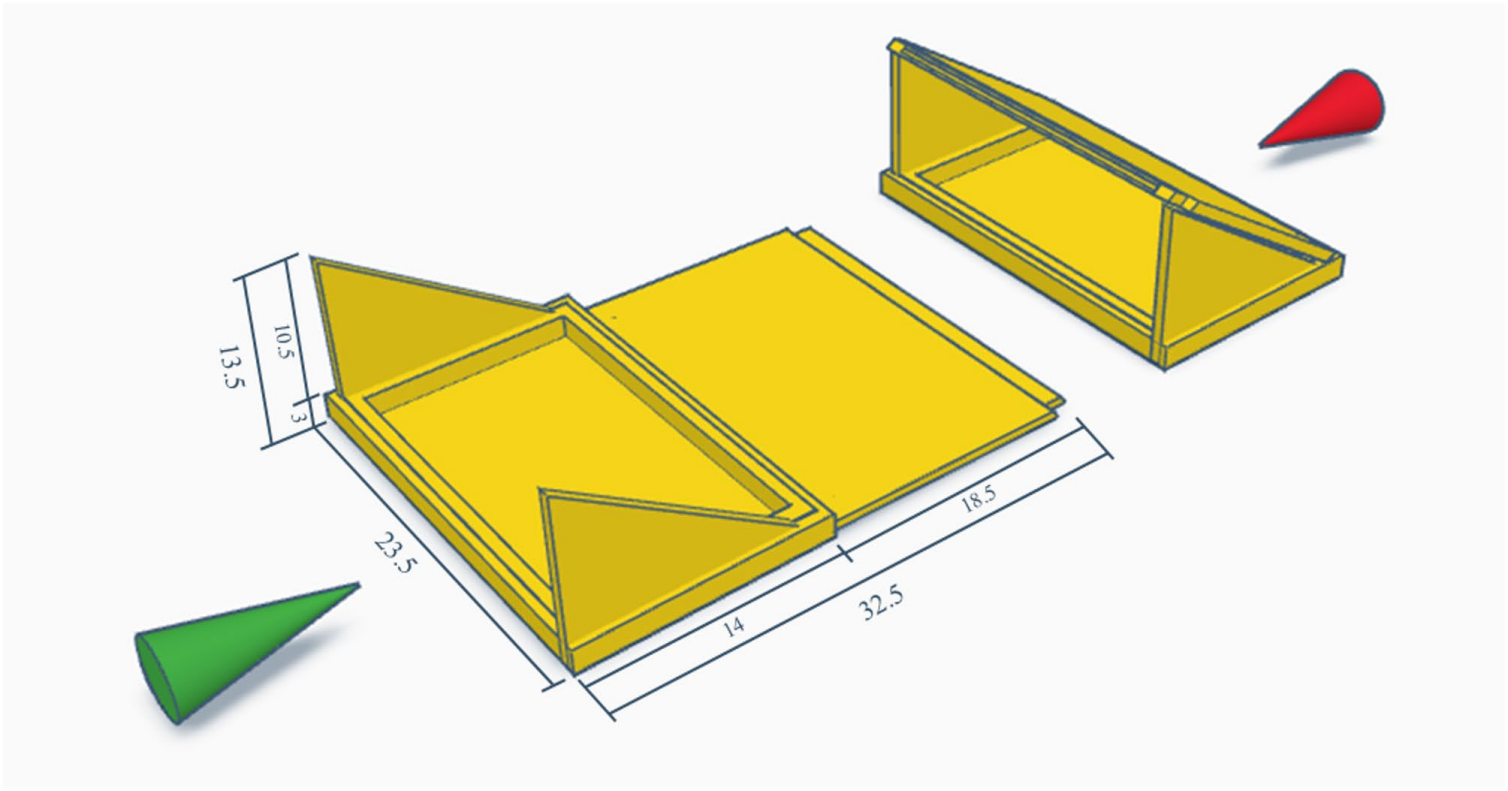
Diagram of the containers used in the experiment, when open and closed. When closed, the contents were only visible to the experimenter (green arrow), but not to the test subject (red arrow). Numbers indicate dimensions in cm.

The Combination task (COMB) assessed whether subjects could combine quantities up to 3, resulting in accumulations of up to 5. We used three opaque containers (two vivid yellow containers and one dark green container, as ungulates are mainly dichromatic and can discriminate brightness, saturation and chromatic contrasts between the blue and yellow to green spectra)^109^. When closed, these containers were designed to remain open on the experimenter’s side but closed to the subjects’ view. Therefore, the quantities were never visible for the animal after the initial display, while the experimenter could move items from one container to the other during the manipulation, without touching any container (Fig. 2). As food items we used carrot pieces of the same size (i.e., being approximately 4 cm high and having a similar diameter), a highly liked food for giraffes that helped maintain their motivation to participate in the task.

**Fig. 3:**
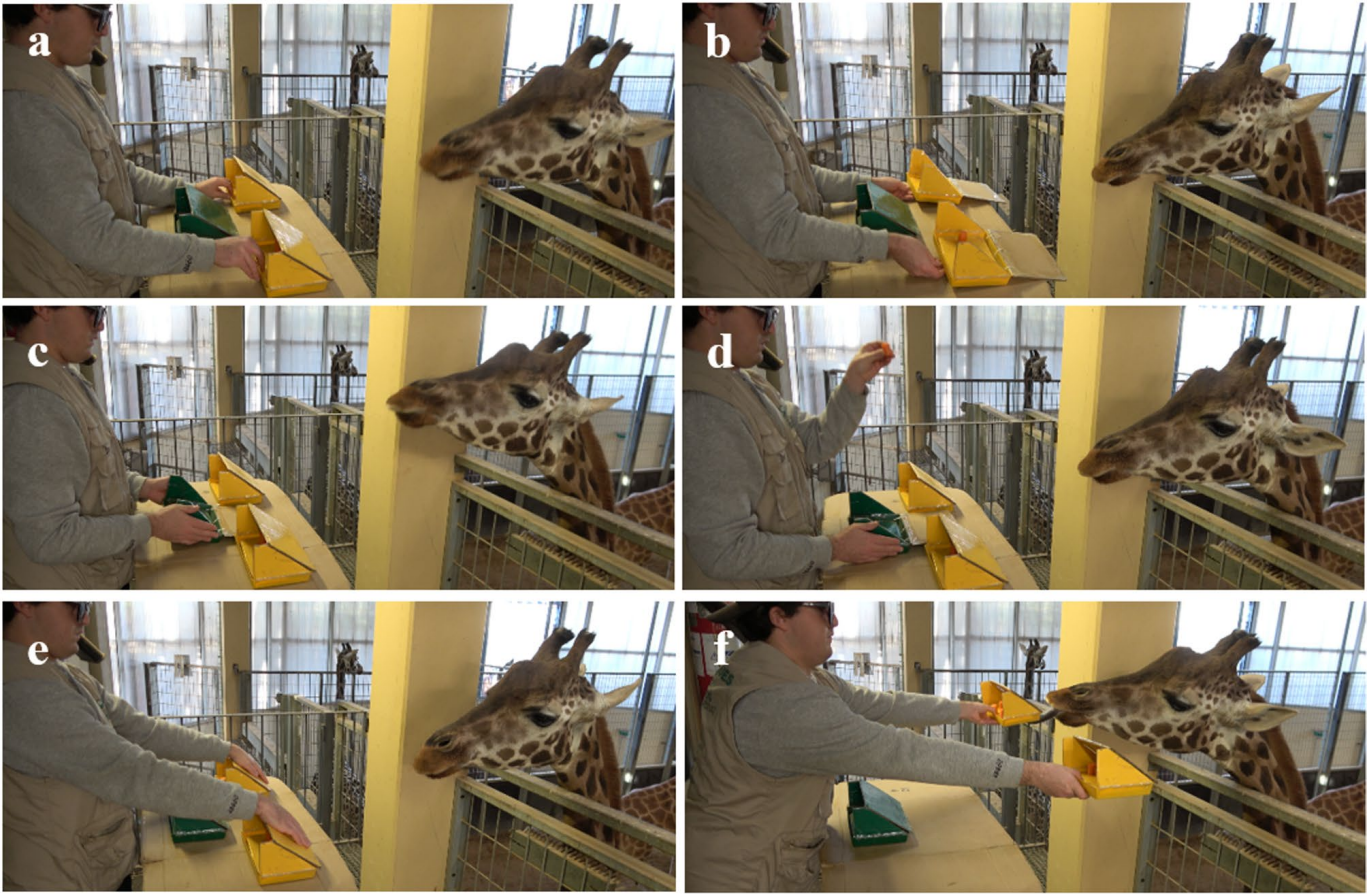
Sequence of a Combination trial. (**a**) After baiting the containers out of the animal’s view, they are placed in front of the subject; (**b**) the yellow containers are opened (5 s), revealing the initial quantities; (**c**) the yellow containers are closed and the green one is opened, showing the amount to be combined (5 s); (**d**) the food pieces are combined one by one; (**e**) both choice options are touched for 5 s to avoid stimulus enhancement; (**f**) the giraffe makes a choice when prompted.

A trial started once the animal was positioned centrally with respect to the setup. The experimenter then baited all the containers out of the subject´s view and placed them closed on the table (90 × 50 × 100 cm) in front of the animal: the two yellow containers on either side (with 50 cm of separation), and the green one in the middle. In full view of the animal but out of reach, the experimenter simultaneously opened the two yellow containers, counted silently to 5 s, and then closed them. Next, the experimenter opened the green container, counted silently to 5 s, and visibly transferred its content (1 to 3 items, depending upon the trial condition), one piece at a time and always using the ipsilateral hand, into one of the two yellow containers (i.e., manipulation). After the manipulation, the experimenter briefly touched both yellow containers simultaneously (to reduce stimulus enhancement toward the last option being manipulated), counted silently to 5 s, and finally allowed the subject to choose between the two yellow containers by carefully and simultaneously lifting them, while leaving the green container on the table (Fig. 3). The animal made a choice by touching one container with its muzzle or tongue and was then allowed to consume the selected quantity. If the selected container corresponded to the option containing the larger quantity, the response was coded as correct (1); otherwise, it was coded as incorrect (0). The animal was given 15 s to make a selection. In the only two instances in which the animal made no choice within this time, the trial was started anew. We tested 18 different conditions, in which just one option was manipulated (Table 3). In 14 of these conditions, the initial quantities were different from zero. However, in 4 conditions, one of the yellow containers started out empty.

**Table 3:** Conditions tested by each task. In the three experimental tasks, the first two numbers represent the initial visible quantities, the sign specifies the manipulation (i.e., + for combinations, – for dissociations, and ± for subsequent operations), the number following the sign the quantity being accumulated/removed, and the last two numbers represent the final quantities. In both control tasks, the two numbers represent the initial visible quantities.

Conditions by task			
Combination	Dissociation	Subsequent events	Visible open and closed
0:2 + 1 = 1:2	1:2 - 1 = 0:2		
2:3 + 1 = 2:4	2:4 - 1 = 2:3		
2:4 + 1 = 2:5	2:5 - 1 = 2:4		
1:3 + 1 = 2:3	2:3 - 1 = 1:3		
2:4 + 1 = 3:4	3:4 - 1 = 2:4		0:1
3:5 + 1 = 4:5	4:5 - 1 = 3:5		0:2
			0:4
0:1 + 2 = 2:1	1:2 - 2 = 1:0	1:4 ± 1 = 2:3	1:5
2:3 + 2 = 2:5	2:5 - 2 = 2:3	1:4 ± 2 = 3:2	1:4
1:2 + 2 = 3:2	3:2 - 2 = 1:2	1:4 ± 3 = 4:1	1:3
1:4 + 2 = 3:4	3:4 - 2 = 1:4	0:5 ± 2 = 2:3	2:5
2:3 + 2 = 4:3	4:3 - 2 = 2:3	0:5 ± 3 = 3:2	1:2
2:5 + 2 = 4:5	4:5 - 2 = 2:5	2:3 ± 1 = 3:2	2:4
3:4 + 2 = 5:4	4:5 - 2 = 4:3		3:5
			2:3
0:2 + 3 = 3:2	2:3 - 3 = 2:0		3:4
0:4 + 3 = 3:4	3:4 - 3 = 0:4		4:5
1:3 + 3 = 4:3	3:4 - 3 = 3:1		
1:5 + 3 = 4:5	4:5 - 3 = 1:5		
2:4 + 3 = 5:4	4:5 - 3 = 4:2		

The Dissociation (DISS) task assessed whether subjects could dissociate up to 3 items from quantities of up to 5. The general procedure was identical to that used in the Combination task, with one key difference: the central green container was initially empty and served as the receptacle for the items removed from the yellow containers. Thus, during the manipulation, the items were transferred from one of the yellow containers into the green one using the ipsilateral hand. We tested the 18 inverse conditions of the combinations (Table 3).

The Subsequent Events (SUBE) task assessed whether subjects could sequentially process a dissociation and a combination when food items were transferred between containers. Outside the subject’s view, the experimenter baited the two yellow containers and closed them before placing them on the table, along with the empty central green container. Once all containers were positioned in front of the subject but still out of reach, the experimenter simultaneously opened both yellow containers, counted silently to 5 s, and then closed them. The experimenter then removed a specific number of items, one by one while using the ipsilateral hand, from one of the yellow containers and placed them into the green one, making them visible to the subject for 5 s while in the green container. These items were then transferred one at a time with the ipsilateral hand (now the hand opposite to the one used in the first step) into the other yellow container. Finally, the experimenter simultaneously touched both yellow containers, counted silently to 5 s, and then presented them closed to the subject for a choice. We included 6 different conditions in this task, two of which started with one empty yellow container (Table 3).

We further added two control tasks to assess the impact of visibility on subjects’ performance. In these tasks, we tested the giraffes’ ability to discriminate between quantities in the absence of any manipulation of the initial sets, that is, no arithmetic-like reasoning was required to succeed. As no manipulation was needed, we only used the two yellow containers. In the Visible Open (VO) task, the experimenter baited the two containers out of the subject’s view, then closed them and placed them on the table, out of the subject’s reach. In full view of the subject, the experimenter opened them for 5 s before allowing the giraffe to choose, by simultaneously lifting both containers with his hands. In the Visible Closed (VC) task, we used exactly the same procedure, but before allowing the giraffe to choose one container, the experimenter closed the containers, so that the quantities were no longer visible at the time of decision. Thus, while VO allowed direct visual comparison at the moment of choice, VC required the animals to rely on their memory, similarly to what happened in the three experimental tasks. The inclusion of these conditions therefore allowed us to disentangle the effect of visibility and manipulation on subjects’ performance. For each of the two control tasks, we tested 13 different conditions (Table 3). The quantities used in these conditions corresponded to the final quantities presented in the COMB and DISS tasks.

Each subject completed four experimental sessions. Each session included one trial for each of the 13 conditions of the VO and VC tasks, the 18 conditions of the COMB and DISS tasks, and the 6 conditions of the SUBE tasks. This resulted in a total of 68 trials per session and 272 trials per subject across the entire experiment (1.088 in total). The order of tasks within each session was counterbalanced across subjects but remained fixed for each individual. Although multiple subjects could be tested on the same day, no subject completed more than one session per day. Within each session, trials were selected to ensure that: (i) we included all possible conditions for each task; (ii) the larger final quantity appeared equally on the left and right sides; and (iii) the correct option did not appear on the same side more than three times in a row. During all sessions, the experimenter wore sunglasses to avoid the Clever Hans effect^110^, while looking down and maintaining neutral facial and body expressions. All trials were recorded using a camera located approximately 1.5 m from the giraffe, at the back of the experimenter, in order to allow for subsequent recoding. We used the package “irr” in R^111^ to assess inter-observer reliability on a sample of 220 trials (i.e., > 20% of the dataset). Agreement between the two raters was excellent, with Cohen’s κ = 1 (*z* = 14.8, *p* < 0.001).

In cases where a side bias emerged (i.e., the animal consistently selected the same side, resulting in three consecutive incorrect choices), we interrupted the session and administered three “side trials”. In these trials, only one yellow container was placed on the side the animal had not been choosing. The container was opened in front of the subject but out of reach. Then, the experimenter placed a single piece of food inside, counted silently to 5 s, closed the container, and allowed the animal to choose between the container and an empty hand. If the animal selected the container in at least two out of the three side trials, the session was resumed from the point it had been paused. If not, the session was discontinued and resumed on the next testing day. This procedure was motivated on evidence indicating that visual side biases are common in artiodactyls and can influence attention to competing options^112^, as observed in other ungulates under similar experimental conditions^107^. Increasing the proportion of trials presented on the non-preferred side when a consistent side bias is detected has been shown to reduce lateral bias and improve performance in choice tasks^113^. Throughout the 1088 trials of the experiment, we applied this procedure 6 times for Nakuru, 8 for Nuru, 8 for Njano and 9 for Yalinga. None of the subjects exhibited lateralized behaviours in the subsequent trials.

In the tasks of this study, subjects had to make their decisions based on mental representations, rather than direct visual perception, because the final quantities were not visible to the subject. Moreover, compared to other approaches, our approach offered several advantages: (1) it imposed a lower working memory load compared to other approaches that involved adding more than three items to the initial quantities and/or manipulating both options (while there is no direct evidence regarding working memory capacities in giraffes, there is evidence that they can remember the location of a reward for up to 30 s^108^, which exceeds the duration of each trial under this protocol); (2) it did not require prior learning or training, such as associating symbols with quantities; and (3) it allowed for the assessment of simpler strategies (i.e., DMO, SLQ and ILQ) that could account for general success in the tasks through simpler cognitive processes.

### Statistical analyses

We ran two generalized linear mixed models in R (R Core Team, version 4.2.2) using the “glmmTMB” package^114^. In the first model (Model 1), we used a binomial regression to test whether the log odds of selecting the larger quantity of food (modelled via a logit link) varied across tasks. In the full model, we included task as predictor, and we controlled for the side of the correct option (to account for possible side biases), session number (as individual performance might increase or decrease through time, because of learning effects or decreasing motivation) and set ratio (as performance in quantity discrimination tasks is known to decrease with increasing set ratio, also for giraffes)^48,95^. Moreover, we included subject identity as random effect, as the same individuals were tested across multiple tasks and conditions. This full model was then compared with likelihood ratio tests to a null model that included only control variables and random effects. In case of a significant difference, we ran a variable drop analysis. If a categorical test predictor was significant, we further conducted post hoc pairwise comparisons to detect significant differences between the levels of the predictor. Residual diagnostics and overdispersion were not an issue, when checked with the “DHARMa” package^115^. Multicollinearity was assessed with the “performance” package^116^, showing a maximum Variance Inflation Factor (VIF) of 1.39, suggesting no significant collinearity.

To assess whether subjects relied on simpler strategies to solve the tasks, we ran a second model (Model 2), after removing the two control tasks (VO and VC) from the dataset (as no simpler strategies could be used to solve these tasks). As for Model 1, we modelled the log odds of selecting the larger quantity of food, using a logit link. As test predictors, we included the task, the number of items moved during the manipulation, and whether the specific trial could be also solved by using simpler strategies (DMO, ILQ, or SLQ). As in Model 1, we further included session number, side of the correct option and set ratio (both initial and final, given that the manipulation modified the comparison) as controls, and subject identity as random effect. We then compared the full model to a null model using likelihood ratio tests and, in case of a significant difference, we ran a variable drop analysis. Residual diagnostics and overdispersion checks showed no issues, but multicollinearity analysis revealed a high correlation between ILQ and SLQ variables. After removing ILQ, the maximum VIF dropped to 2.93, indicating no significant collinearity.

Finally, for each subject and task, we assessed performance at the individual level with binomial proportion tests, to assess if the proportion of correct choices significantly deviated from chance (50%). This approach allowed for a more accurate interpretation of the variation observed in the models, as significant differences between tasks do not necessarily imply performance above chance. If Model 2 revealed a significant effect of DMO or SLQ (suggesting that subjects relied on simpler strategies to solve the task), we reran the binomial tests after splitting the dataset in two: one with the trials that could be solved using this strategy, and one with the trials that could not be solved with this strategy. Given that, to avoid learning effects, we administered a low number of trials per condition in each task, we assessed reliance on simpler strategies across the entire dataset, rather than separately for each task, although future studies should ideally disentangle whether there is variation, across tasks and trial types, in the use of different alternative strategies. Statistical significance was set at α = 0.05 for all tests.

## Supplementary Information

Below is the link to the electronic supplementary material.

Supplementary Material 1

Supplementary Material 2

Supplementary Material 3

Supplementary Material 4

Supplementary Material 5

## Data Availability

The datasets generated and analyzed during the current study are available as Supplementary Material.
