# Supplementary material for: Assessing quantity combination and dissociation in giraffes
Source: Sci Rep. 2026 Jun 26;16:19615. doi: 10.1038/s41598-026-54126-7 (PMC13309573; doi:10.1038/s41598-026-54126-7)
Supplement: Supplementary file 3 — Supplementary Material 3 [file 41598_2026_54126_MOESM3_ESM.docx]

**
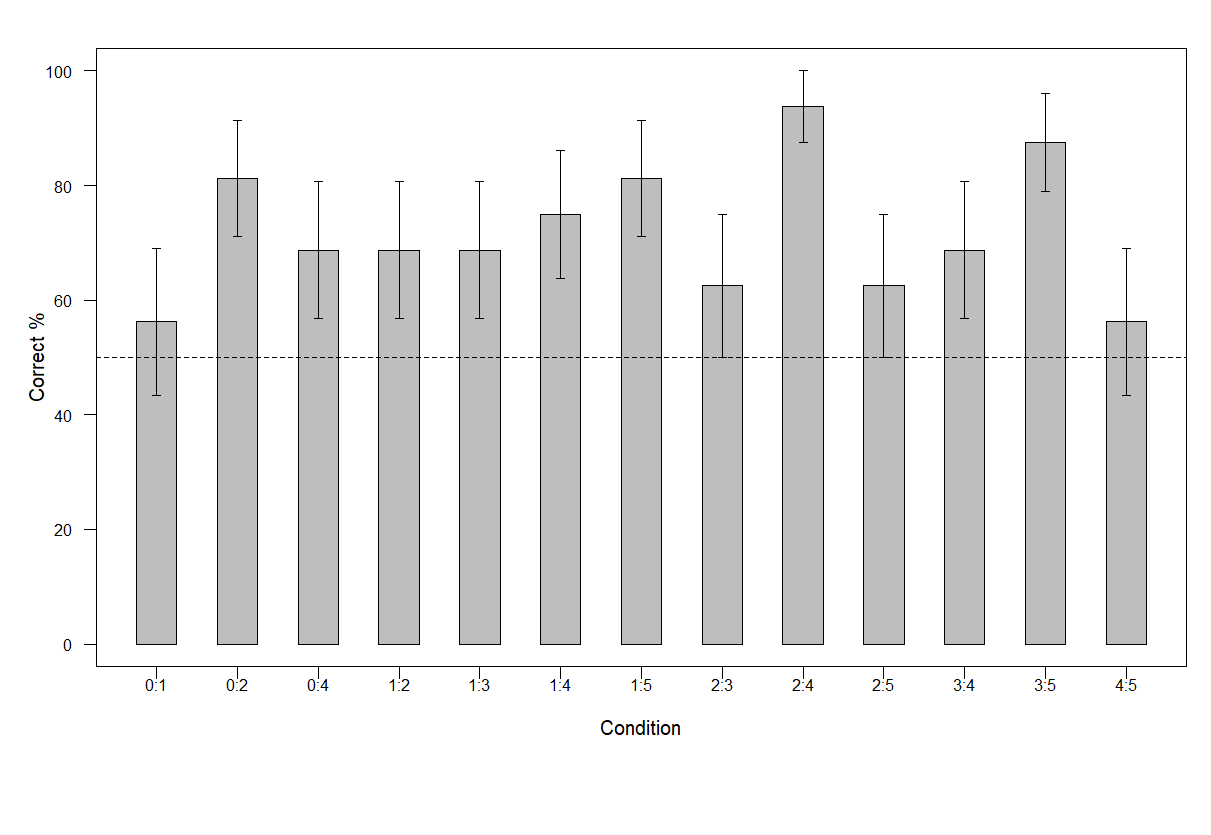
Percentage of correct responses per task and condition**

**Supplementary Figure S1**. **Percentage of correct responses by Condition in the Visual Open (VO) task.** The tops of the bars indicate the mean, and the whiskers denote the standard error (±SE). This graph should be considered purely descriptive, as the number of trials administered for each condition was too low (N = 4 per individual) to allow statistical inference across conditions.


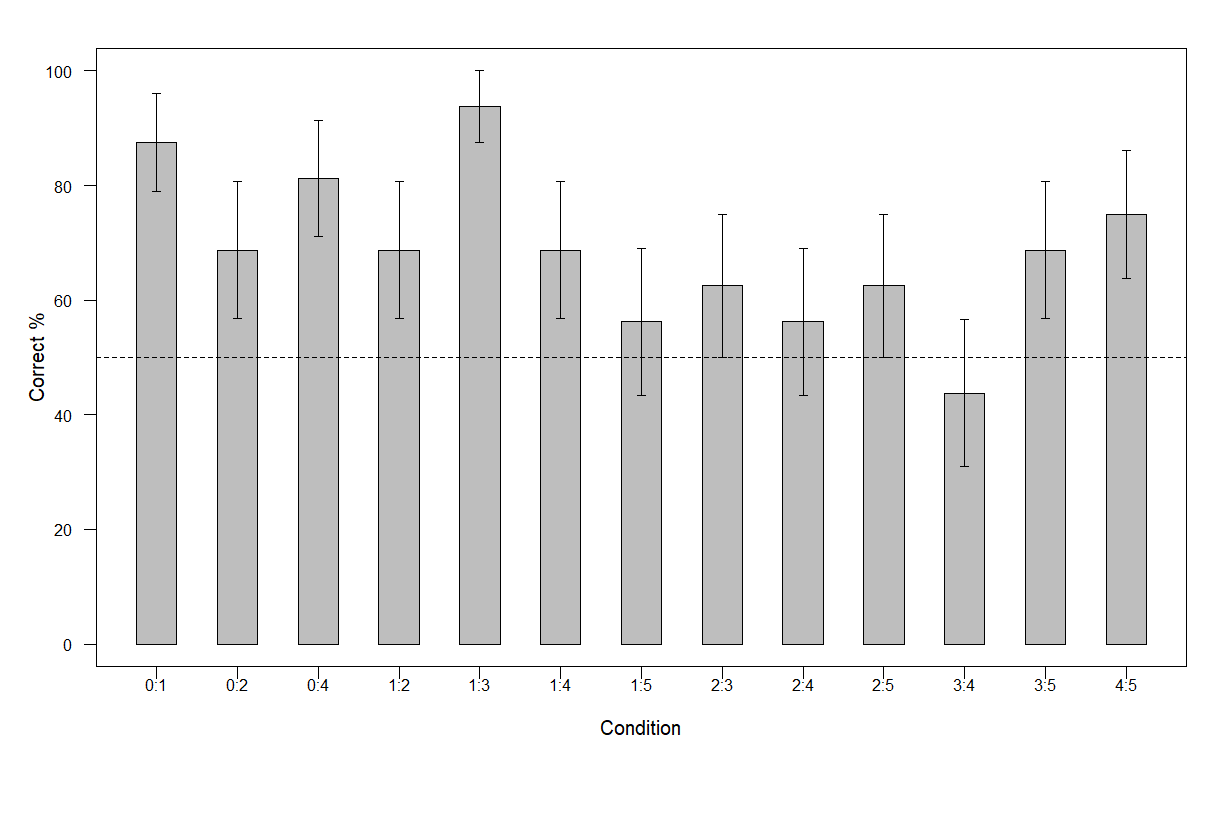


**Supplementary Figure S2**. **Percentage of correct responses by Condition in the Visual Closed (VC) task.** The tops of the bars indicate the mean, and the whiskers denote the standard error (±SE). This graph should be considered purely descriptive, as the number of trials administered for each condition was too low (N = 4 per individual) to allow statistical inference across conditions.


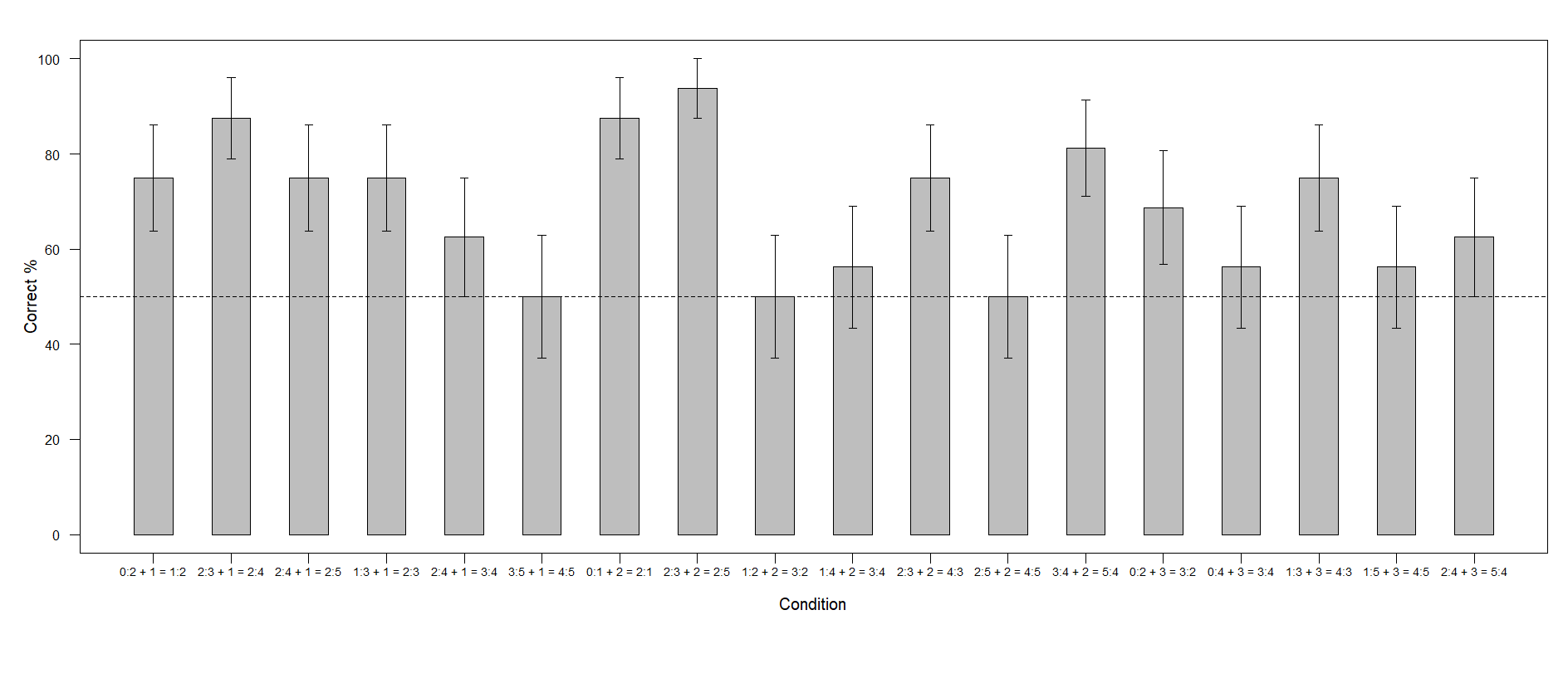


**Supplementary Figure S3**. **Percentage of correct responses by Condition in the Combination (COMB) task.** The tops of the bars indicate the mean, and the whiskers denote the standard error (±SE). This graph should be considered purely descriptive, as the number of trials administered for each condition was too low (N = 4 per individual) to allow statistical inference across conditions.


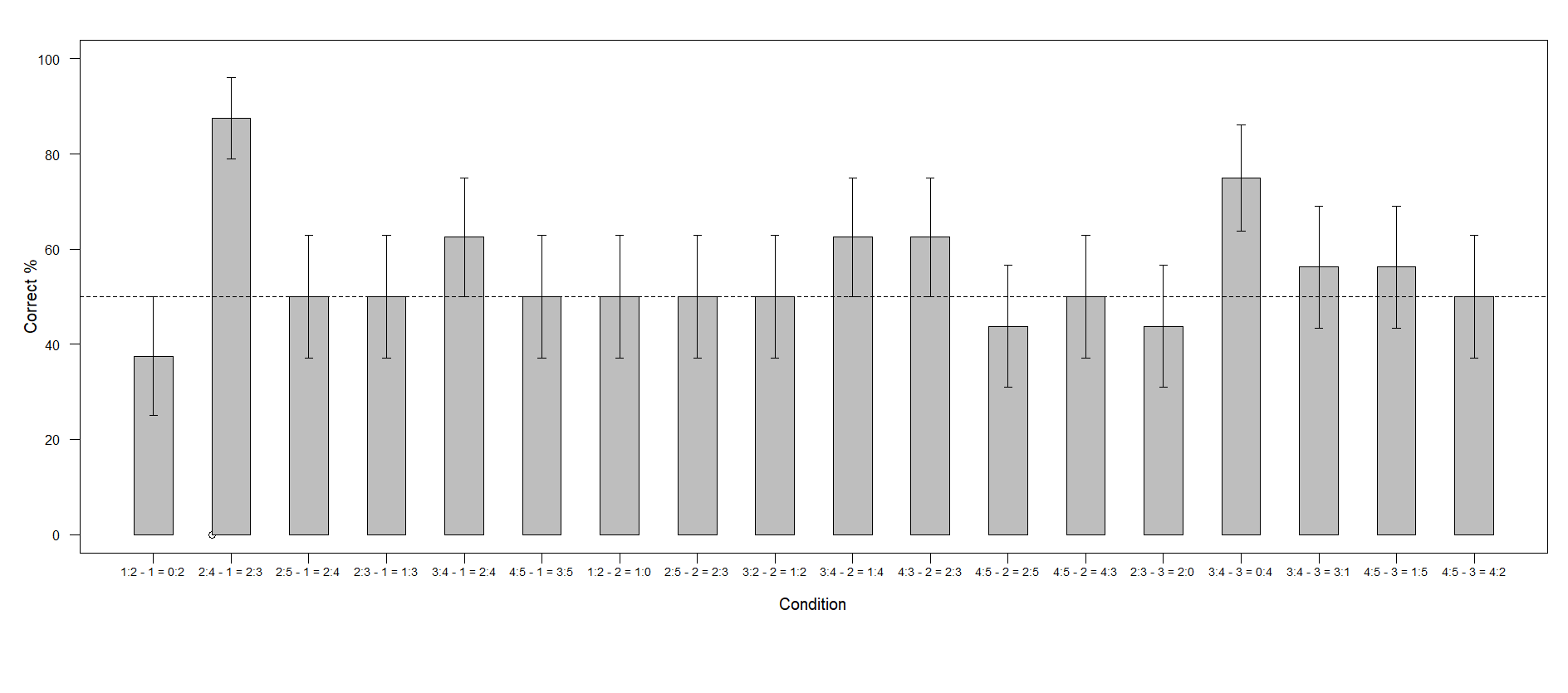


**Supplementary Figure S4**. **Percentage of correct responses by Condition in the Dissociation (DISS) task.** The tops of the bars indicate the mean, and the whiskers denote the standard error (±SE). This graph should be considered purely descriptive, as the number of trials administered for each condition was too low (N = 4 per individual) to allow statistical inference across conditions.


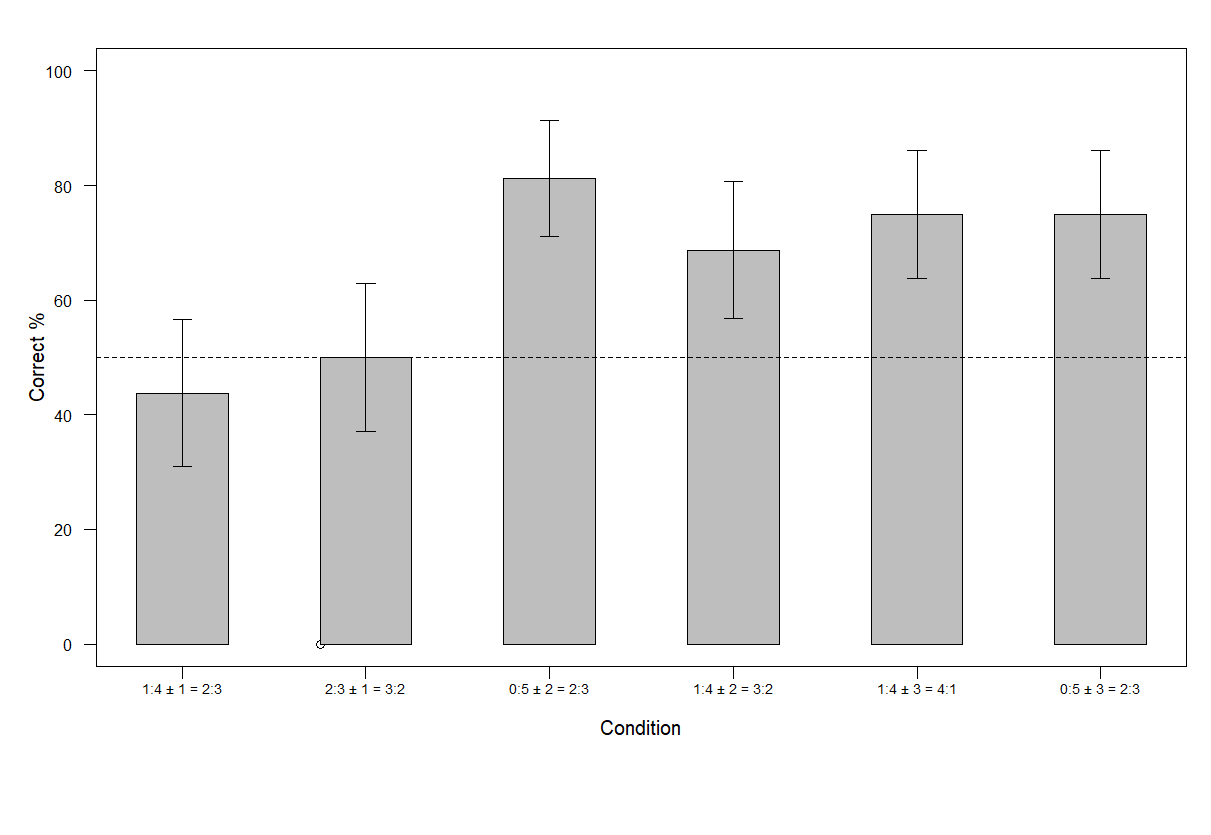


**Supplementary Figure S5**. **Percentage of correct responses by Condition in the Subsequent Events (SUBE) task.** The tops of the bars indicate the mean, and the whiskers denote the standard error (±SE). This graph should be considered purely descriptive, as the number of trials administered for each condition was too low (N = 4 per individual) to allow statistical inference across conditions.

**Performance per task, individual and condition**

**Table S1. Individual performance as a function of Condition in Visual Open (VO) task.** Includes individual number of correct responses (Correct / total of 4), probability of success (Probability), the standard error (SE) and the confidence interval (CI).

| **Subject** | **Condition** | **Correct** | **Probability** | **SE** | **2.5 - 97.5% CI** |
| --- | --- | --- | --- | --- | --- |
| **Nakuru** | 0vs1 | 2 | 50.0 | 25.0 | 1.0 - 99.0 |
|  | 0vs2 | 4 | 100.0 | 0.0 | 100.0 |
|  | 0vs4 | 4 | 100.0 | 0.0 | 100.0 |
|  | 1vs2 | 3 | 75.0 | 21.7 | 32.6 - 100.0 |
|  | 1vs3 | 3 | 75.0 | 21.7 | 32.6 - 100.0 |
|  | 1vs4 | 2 | 50.0 | 25.0 | 1.0 - 99.0 |
|  | 1vs5 | 2 | 50.0 | 25.0 | 1.0 - 99.0 |
|  | 2vs3 | 2 | 50.0 | 25.0 | 1.0 - 99.0 |
|  | 2vs4 | 4 | 100.0 | 0.0 | 100.0 |
|  | 2vs5 | 4 | 100.0 | 0.0 | 100.0 |
|  | 3vs4 | 1 | 25.0 | 21.7 | 0.0 - 67.4 |
|  | 3vs5 | 4 | 100.0 | 0.0 | 100.0 |
|  | 4vs5 | 2 | 50.0 | 25.0 | 1.0 - 99.0 |
|  |  |  |  |  |  |
| **Njano** | 0vs1 | 2 | 50.0 | 25.0 | 1.0 - 99.0 |
|  | 0vs2 | 3 | 75.0 | 21.7 | 32.6 - 100.0 |
|  | 0vs4 | 2 | 50.0 | 25.0 | 1.0 - 99.0 |
|  | 1vs2 | 3 | 75.0 | 21.7 | 32.6 - 100.0 |
|  | 1vs3 | 3 | 75.0 | 21.7 | 32.6 - 100.0 |
|  | 1vs4 | 3 | 75.0 | 21.7 | 32.6 - 100.0 |
|  | 1vs5 | 4 | 100.0 | 0.0 | 100.0 |
|  | 2vs3 | 3 | 75.0 | 21.7 | 32.6 - 100.0 |
|  | 2vs4 | 3 | 75.0 | 21.7 | 32.6 - 100.0 |
|  | 2vs5 | 1 | 25.0 | 21.7 | 0.0 - 67.4 |
|  | 3vs4 | 4 | 100.0 | 0.0 | 100.0 |
|  | 3vs5 | 3 | 75.0 | 21.7 | 32.6 - 100.0 |
|  | 4vs5 | 2 | 50.0 | 25.0 | 1.0 - 99.0 |
|  |  |  |  |  |  |
| **Nuru** | 0vs1 | 3 | 75.0 | 21.7 | 32.6 - 100.0 |
|  | 0vs2 | 2 | 50.0 | 25.0 | 1.0 - 99.0 |
|  | 0vs4 | 3 | 75.0 | 21.7 | 32.6 - 100.0 |
|  | 1vs2 | 1 | 25.0 | 21.7 | 0.0 - 67.4 |
|  | 1vs3 | 2 | 50.0 | 25.0 | 1.0 - 99.0 |
|  | 1vs4 | 4 | 100.0 | 0.0 | 100.0 |
|  | 1vs5 | 4 | 100.0 | 0.0 | 100.0 |
|  | 2vs3 | 2 | 50.0 | 25.0 | 1.0 - 99.0 |
|  | 2vs4 | 4 | 100.0 | 0.0 | 100.0 |
|  | 2vs5 | 3 | 75.0 | 21.7 | 32.6 - 100.0 |
|  | 3vs4 | 4 | 100.0 | 0.0 | 100.0 |
|  | 3vs5 | 4 | 100.0 | 0.0 | 100.0 |
|  | 4vs5 | 3 | 75.0 | 21.7 | 32.6 - 100.0 |
|  |  |  |  |  |  |
| **Yalinga** | 0vs1 | 2 | 50.0 | 25.0 | 1.0 - 99.0 |
|  | 0vs2 | 4 | 100.0 | 0.0 | 100.0 |
|  | 0vs4 | 2 | 50.0 | 25.0 | 1.0 - 99.0 |
|  | 1vs2 | 4 | 100.0 | 0.0 | 100.0 |
|  | 1vs3 | 3 | 75.0 | 21.7 | 32.6 - 100.0 |
|  | 1vs4 | 3 | 75.0 | 21.7 | 32.6 - 100.0 |
|  | 1vs5 | 3 | 75.0 | 21.7 | 32.6 - 100.0 |
|  | 2vs3 | 3 | 75.0 | 21.7 | 32.6 - 100.0 |
|  | 2vs4 | 4 | 100.0 | 0.0 | 100.0 |
|  | 2vs5 | 2 | 50.0 | 25.0 | 1.0 - 99.0 |
|  | 3vs4 | 2 | 50.0 | 25.0 | 1.0 - 99.0 |
|  | 3vs5 | 3 | 75.0 | 21.7 | 32.6 - 100.0 |
|  | 4vs5 | 2 | 50.0 | 25.0 | 1.0 - 99.0 |

**Table S2. Individual performance as a function of Condition in Visual Closed (VO) task.** Includes individual number of correct responses (Correct / total of 4), probability of success (Probability), the standard error (SE) and the confidence interval (CI).

| **Subject** | **Condition** | **Correct** | **Probability** | **SE** | **2.5 - 97.5% CI** |
| --- | --- | --- | --- | --- | --- |
| **Nakuru** | 0vs1 | 4 | 100.0 | 0.0 | 100.0 |
|  | 0vs2 | 2 | 50.0 | 25.0 | 1.0 - 99.0 |
|  | 0vs4 | 4 | 100.0 | 0.0 | 100.0 |
|  | 1vs2 | 3 | 75.0 | 21.7 | 32.6 - 100.0 |
|  | 1vs3 | 4 | 100.0 | 0.0 | 100.0 |
|  | 1vs4 | 3 | 75.0 | 21.7 | 32.6 - 100.0 |
|  | 1vs5 | 2 | 50.0 | 25.0 | 1.0 - 99.0 |
|  | 2vs3 | 1 | 25.0 | 21.7 | 0.0 - 67.4 |
|  | 2vs4 | 2 | 50.0 | 25.0 | 1.0 - 99.0 |
|  | 2vs5 | 2 | 50.0 | 25.0 | 1.0 - 99.0 |
|  | 3vs4 | 1 | 25.0 | 21.7 | 0.0 - 67.4 |
|  | 3vs5 | 3 | 75.0 | 21.7 | 32.6 - 100.0 |
|  | 4vs5 | 3 | 75.0 | 21.7 | 32.6 - 100.0 |
|  |  |  |  |  |  |
| **Njano** | 0vs1 | 3 | 75.0 | 21.7 | 32.6 - 100.0 |
|  | 0vs2 | 3 | 75.0 | 21.7 | 32.6 - 100.0 |
|  | 0vs4 | 3 | 75.0 | 21.7 | 32.6 - 100.0 |
|  | 1vs2 | 3 | 75.0 | 21.7 | 32.6 - 100.0 |
|  | 1vs3 | 4 | 100.0 | 0.0 | 100.0 |
|  | 1vs4 | 2 | 50.0 | 25.0 | 1.0 - 99.0 |
|  | 1vs5 | 3 | 75.0 | 21.7 | 32.6 - 100.0 |
|  | 2vs3 | 4 | 100.0 | 0.0 | 100.0 |
|  | 2vs4 | 2 | 50.0 | 25.0 | 1.0 - 99.0 |
|  | 2vs5 | 3 | 75.0 | 21.7 | 32.6 - 100.0 |
|  | 3vs4 | 0 | 0.0 | 0.0 | 0.0 - 67.4 |
|  | 3vs5 | 2 | 50.0 | 25.0 | 1.0 - 99.0 |
|  | 4vs5 | 3 | 75.0 | 21.7 | 32.6 - 100.0 |
|  |  |  |  |  |  |
| **Nuru** | 0vs1 | 4 | 100.0 | 0.0 | 100.0 |
|  | 0vs2 | 2 | 50.0 | 25.0 | 1.0 - 99.0 |
|  | 0vs4 | 3 | 75.0 | 21.7 | 32.6 - 100.0 |
|  | 1vs2 | 3 | 75.0 | 21.7 | 32.6 - 100.0 |
|  | 1vs3 | 3 | 75.0 | 21.7 | 32.6 - 100.0 |
|  | 1vs4 | 3 | 75.0 | 21.7 | 32.6 - 100.0 |
|  | 1vs5 | 1 | 25.0 | 21.7 | 0.0 - 67.4 |
|  | 2vs3 | 1 | 25.0 | 21.7 | 0.0 - 67.4 |
|  | 2vs4 | 2 | 50.0 | 25.0 | 1.0 - 99.0 |
|  | 2vs5 | 2 | 50.0 | 25.0 | 1.0 - 99.0 |
|  | 3vs4 | 3 | 75.0 | 21.7 | 32.6 - 100.0 |
|  | 3vs5 | 2 | 50.0 | 25.0 | 1.0 - 99.0 |
|  | 4vs5 | 3 | 75.0 | 21.7 | 32.6 - 100.0 |
|  |  |  |  |  |  |
| **Yalinga** | 0vs1 | 3 | 75.0 | 21.7 | 32.6 - 100.0 |
|  | 0vs2 | 4 | 100.0 | 0.0 | 100.0 |
|  | 0vs4 | 3 | 75.0 | 21.7 | 32.6 - 100.0 |
|  | 1vs2 | 2 | 50.0 | 25.0 | 1.0 - 99.0 |
|  | 1vs3 | 4 | 100.0 | 0.0 | 100.0 |
|  | 1vs4 | 3 | 75.0 | 21.7 | 32.6 - 100.0 |
|  | 1vs5 | 3 | 75.0 | 21.7 | 32.6 - 100.0 |
|  | 2vs3 | 4 | 100.0 | 0.0 | 100.0 |
|  | 2vs4 | 3 | 75.0 | 21.7 | 32.6 - 100.0 |
|  | 2vs5 | 3 | 75.0 | 21.7 | 32.6 - 100.0 |
|  | 3vs4 | 3 | 75.0 | 21.7 | 32.6 - 100.0 |
|  | 3vs5 | 4 | 100.0 | 0.0 | 100.0 |
|  | 4vs5 | 3 | 75.0 | 21.7 | 32.6 - 100.0 |

**Table S3. Individual performance as a function of Condition in Combination (COMB) task.** Includes individual number of correct responses (Correct / total of 4), probability of success (Probability), the standard error (SE) and the confidence interval (CI).

| **Subject** | **Condition** | **Correct** | **Probability** | **SE** | **2.5 - 97.5% CI** |
| --- | --- | --- | --- | --- | --- |
| **Nakuru** | 0:2 + 1 = 1:2 | 3 | 75.0 | 21.7 | 32.6 - 100.0 |
|  | 2:3 + 1 = 2:4 | 4 | 100.0 | 0.0 | 100.0 |
|  | 2:4 + 1 = 2:5 | 3 | 75.0 | 21.7 | 32.6 - 100.0 |
|  | 1:3 + 1 = 2:3 | 3 | 75.0 | 21.7 | 32.6 - 100.0 |
|  | 2:4 + 1 = 3:4 | 2 | 50.0 | 25.0 | 1.0 - 99.0 |
|  | 3:5 + 1 = 4:5 | 1 | 25.0 | 21.7 | 0.0 - 67.4 |
|  | 0:1 + 2 = 2:1 | 4 | 100.0 | 0.0 | 100.0 |
|  | 2:3 + 2 = 2:5 | 4 | 100.0 | 0.0 | 100.0 |
|  | 1:2 + 2 = 3:2 | 1 | 25.0 | 21.7 | 0.0 - 67.4 |
|  | 1:4 + 2 = 3:4 | 2 | 50.0 | 25.0 | 1.0 - 99.0 |
|  | 2:3 + 2 = 4:3 | 3 | 75.0 | 21.7 | 32.6 - 100.0 |
|  | 2:5 + 2 = 4:5 | 2 | 50.0 | 25.0 | 1.0 - 99.0 |
|  | 3:4 + 2 = 5:4 | 4 | 100.0 | 0.0 | 100.0 |
|  | 0:2 + 3 = 3:2 | 3 | 75.0 | 21.7 | 32.6 - 100.0 |
|  | 0:4 + 3 = 3:4 | 3 | 75.0 | 21.7 | 32.6 - 100.0 |
|  | 1:3 + 3 = 4:3 | 3 | 75.0 | 21.7 | 32.6 - 100.0 |
|  | 1:5 + 3 = 4:5 | 1 | 25.0 | 21.7 | 0.0 - 67.4 |
|  | 2:4 + 3 = 5:4 | 3 | 75.0 | 21.7 | 32.6 - 100.0 |
|  |  |  |  |  |  |
| **Njano** | 0:2 + 1 = 1:2 | 2 | 50.0 | 25.0 | 1.0 - 99.0 |
|  | 2:3 + 1 = 2:4 | 4 | 100.0 | 0.0 | 100.0 |
|  | 2:4 + 1 = 2:5 | 3 | 75.0 | 21.7 | 32.6 - 100.0 |
|  | 1:3 + 1 = 2:3 | 4 | 100.0 | 0.0 | 100.0 |
|  | 2:4 + 1 = 3:4 | 2 | 50.0 | 25.0 | 1.0 - 99.0 |
|  | 3:5 + 1 = 4:5 | 2 | 50.0 | 25.0 | 1.0 - 99.0 |
|  | 0:1 + 2 = 2:1 | 4 | 100.0 | 0.0 | 100.0 |
|  | 2:3 + 2 = 2:5 | 3 | 75.0 | 21.7 | 32.6 - 100.0 |
|  | 1:2 + 2 = 3:2 | 3 | 75.0 | 21.7 | 32.6 - 100.0 |
|  | 1:4 + 2 = 3:4 | 2 | 50.0 | 25.0 | 1.0 - 99.0 |
|  | 2:3 + 2 = 4:3 | 3 | 75.0 | 21.7 | 32.6 - 100.0 |
|  | 2:5 + 2 = 4:5 | 2 | 50.0 | 25.0 | 1.0 - 99.0 |
|  | 3:4 + 2 = 5:4 | 2 | 50.0 | 25.0 | 1.0 - 99.0 |
|  | 0:2 + 3 = 3:2 | 2 | 50.0 | 25.0 | 1.0 - 99.0 |
|  | 0:4 + 3 = 3:4 | 3 | 75.0 | 21.7 | 32.6 - 100.0 |
|  | 1:3 + 3 = 4:3 | 2 | 50.0 | 25.0 | 1.0 - 99.0 |
|  | 1:5 + 3 = 4:5 | 3 | 75.0 | 21.7 | 32.6 - 100.0 |
|  | 2:4 + 3 = 5:4 | 2 | 50.0 | 25.0 | 1.0 - 99.0 |
|  |  |  |  |  |  |
| **Nuru** | 0:2 + 1 = 1:2 | 4 | 100.0 | 0.0 | 100.0 |
|  | 2:3 + 1 = 2:4 | 3 | 75.0 | 21.7 | 32.6 - 100.0 |
|  | 2:4 + 1 = 2:5 | 3 | 75.0 | 21.7 | 32.6 - 100.0 |
|  | 1:3 + 1 = 2:3 | 2 | 50.0 | 25.0 | 1.0 - 99.0 |
|  | 2:4 + 1 = 3:4 | 4 | 100.0 | 0.0 | 100.0 |
|  | 3:5 + 1 = 4:5 | 3 | 75.0 | 21.7 | 32.6 - 100.0 |
|  | 0:1 + 2 = 2:1 | 4 | 100.0 | 0.0 | 100.0 |
|  | 2:3 + 2 = 2:5 | 4 | 100.0 | 0.0 | 100.0 |
|  | 1:2 + 2 = 3:2 | 2 | 50.0 | 25.0 | 1.0 - 99.0 |
|  | 1:4 + 2 = 3:4 | 3 | 75.0 | 21.7 | 32.6 - 100.0 |
|  | 2:3 + 2 = 4:3 | 3 | 75.0 | 21.7 | 32.6 - 100.0 |
|  | 2:5 + 2 = 4:5 | 2 | 50.0 | 25.0 | 1.0 - 99.0 |
|  | 3:4 + 2 = 5:4 | 4 | 100.0 | 0.0 | 100.0 |
|  | 0:2 + 3 = 3:2 | 3 | 75.0 | 21.7 | 32.6 - 100.0 |
|  | 0:4 + 3 = 3:4 | 2 | 50.0 | 25.0 | 1.0 - 99.0 |
|  | 1:3 + 3 = 4:3 | 3 | 75.0 | 21.7 | 32.6 - 100.0 |
|  | 1:5 + 3 = 4:5 | 2 | 50.0 | 25.0 | 1.0 - 99.0 |
|  | 2:4 + 3 = 5:4 | 2 | 50.0 | 25.0 | 1.0 - 99.0 |
|  |  |  |  |  |  |
| **Yalinga** | 0:2 + 1 = 1:2 | 3 | 75.0 | 21.7 | 32.6 - 100.0 |
|  | 2:3 + 1 = 2:4 | 3 | 75.0 | 21.7 | 32.6 - 100.0 |
|  | 2:4 + 1 = 2:5 | 3 | 75.0 | 21.7 | 32.6 - 100.0 |
|  | 1:3 + 1 = 2:3 | 3 | 75.0 | 21.7 | 32.6 - 100.0 |
|  | 2:4 + 1 = 3:4 | 2 | 50.0 | 25.0 | 1.0 - 99.0 |
|  | 3:5 + 1 = 4:5 | 2 | 50.0 | 25.0 | 1.0 - 99.0 |
|  | 0:1 + 2 = 2:1 | 2 | 50.0 | 25.0 | 1.0 - 99.0 |
|  | 2:3 + 2 = 2:5 | 4 | 100.0 | 0.0 | 100.0 |
|  | 1:2 + 2 = 3:2 | 2 | 50.0 | 25.0 | 1.0 - 99.0 |
|  | 1:4 + 2 = 3:4 | 2 | 50.0 | 25.0 | 1.0 - 99.0 |
|  | 2:3 + 2 = 4:3 | 3 | 75.0 | 21.7 | 32.6 - 100.0 |
|  | 2:5 + 2 = 4:5 | 2 | 50.0 | 25.0 | 1.0 - 99.0 |
|  | 3:4 + 2 = 5:4 | 3 | 75.0 | 21.7 | 32.6 - 100.0 |
|  | 0:2 + 3 = 3:2 | 3 | 75.0 | 21.7 | 32.6 - 100.0 |
|  | 0:4 + 3 = 3:4 | 1 | 25.0 | 21.7 | 0.0 - 67.4 |
|  | 1:3 + 3 = 4:3 | 4 | 100.0 | 0.0 | 100.0 |
|  | 1:5 + 3 = 4:5 | 3 | 75.0 | 21.7 | 32.6 - 100.0 |
|  | 2:4 + 3 = 5:4 | 3 | 75.0 | 21.7 | 32.6 - 100.0 |

**Table S4. Individual performance as a function of Condition in Dissociation (DISS) task.** Includes individual number of correct responses (Correct / total of 4), probability of success (Probability), the standard error (SE) and the confidence interval (CI).

| **Subject** | **Condition** | **Correct** | **Probability** | **SE** | **2.5 - 97.5% CI** |
| --- | --- | --- | --- | --- | --- |
| **Nakuru** | 1:2 - 1 = 0:2 | 0 | 0.0 | 0.0 | 0.0 |
|  | 2:4 - 1 = 2:3 | 3 | 75.0 | 21.7 | 32.6 - 100.0 |
|  | 2:5 - 1 = 2:4 | 2 | 50.0 | 25.0 | 1.0 - 99.0 |
|  | 2:3 - 1 = 1:3 | 3 | 75.0 | 21.7 | 32.6 - 100.0 |
|  | 3:4 - 1 = 2:4 | 3 | 75.0 | 21.7 | 32.6 - 100.0 |
|  | 4:5 - 1 = 3:5 | 2 | 50.0 | 25.0 | 1.0 - 99.0 |
|  | 1:2 - 2 = 1:0 | 2 | 50.0 | 25.0 | 1.0 - 99.0 |
|  | 2:5 - 2 = 2:3 | 2 | 50.0 | 25.0 | 1.0 - 99.0 |
|  | 3:2 - 2 = 1:2 | 1 | 25.0 | 21.7 | 0.0 - 67.4 |
|  | 3:4 - 2 = 1:4 | 3 | 75.0 | 21.7 | 32.6 - 100.0 |
|  | 4:3 - 2 = 2:3 | 3 | 75.0 | 21.7 | 32.6 - 100.0 |
|  | 4:5 - 2 = 2:5 | 3 | 75.0 | 21.7 | 32.6 - 100.0 |
|  | 4:5 - 2 = 4:3 | 2 | 50.0 | 25.0 | 1.0 - 99.0 |
|  | 2:3 - 3 = 2:0 | 2 | 50.0 | 25.0 | 1.0 - 99.0 |
|  | 3:4 - 3 = 0:4 | 2 | 50.0 | 25.0 | 1.0 - 99.0 |
|  | 3:4 - 3 = 3:1 | 2 | 50.0 | 25.0 | 1.0 - 99.0 |
|  | 4:5 - 3 = 1:5 | 3 | 75.0 | 21.7 | 32.6 - 100.0 |
|  | 4:5 - 3 = 4:2 | 0 | 0.0 | 0.0 | 0.0 |
|  |  |  |  |  |  |
| **Njano** | 1:2 - 1 = 0:2 | 2 | 50.0 | 25.0 | 1.0 - 99.0 |
|  | 2:4 - 1 = 2:3 | 4 | 100.0 | 0.0 | 100.0 |
|  | 2:5 - 1 = 2:4 | 2 | 50.0 | 25.0 | 1.0 - 99.0 |
|  | 2:3 - 1 = 1:3 | 1 | 25.0 | 21.7 | 0.0 - 67.4 |
|  | 3:4 - 1 = 2:4 | 3 | 75.0 | 21.7 | 32.6 - 100.0 |
|  | 4:5 - 1 = 3:5 | 2 | 50.0 | 25.0 | 1.0 - 99.0 |
|  | 1:2 - 2 = 1:0 | 1 | 25.0 | 21.7 | 0.0 - 67.4 |
|  | 2:5 - 2 = 2:3 | 3 | 75.0 | 21.7 | 32.6 - 100.0 |
|  | 3:2 - 2 = 1:2 | 2 | 50.0 | 25.0 | 1.0 - 99.0 |
|  | 3:4 - 2 = 1:4 | 3 | 75.0 | 21.7 | 32.6 - 100.0 |
|  | 4:3 - 2 = 2:3 | 2 | 50.0 | 25.0 | 1.0 - 99.0 |
|  | 4:5 - 2 = 2:5 | 1 | 25.0 | 21.7 | 0.0 - 67.4 |
|  | 4:5 - 2 = 4:3 | 4 | 100.0 | 0.0 | 100.0 |
|  | 2:3 - 3 = 2:0 | 2 | 50.0 | 25.0 | 1.0 - 99.0 |
|  | 3:4 - 3 = 0:4 | 3 | 75.0 | 21.7 | 32.6 - 100.0 |
|  | 3:4 - 3 = 3:1 | 2 | 50.0 | 25.0 | 1.0 - 99.0 |
|  | 4:5 - 3 = 1:5 | 2 | 50.0 | 25.0 | 1.0 - 99.0 |
|  | 4:5 - 3 = 4:2 | 3 | 75.0 | 21.7 | 32.6 - 100.0 |
|  |  |  |  |  |  |
| **Nuru** | 1:2 - 1 = 0:2 | 2 | 50.0 | 25.0 | 1.0 - 99.0 |
|  | 2:4 - 1 = 2:3 | 3 | 75.0 | 21.7 | 32.6 - 100.0 |
|  | 2:5 - 1 = 2:4 | 3 | 75.0 | 21.7 | 32.6 - 100.0 |
|  | 2:3 - 1 = 1:3 | 2 | 50.0 | 25.0 | 1.0 - 99.0 |
|  | 3:4 - 1 = 2:4 | 3 | 75.0 | 21.7 | 32.6 - 100.0 |
|  | 4:5 - 1 = 3:5 | 2 | 50.0 | 25.0 | 1.0 - 99.0 |
|  | 1:2 - 2 = 1:0 | 3 | 75.0 | 21.7 | 32.6 - 100.0 |
|  | 2:5 - 2 = 2:3 | 1 | 25.0 | 21.7 | 0.0 - 67.4 |
|  | 3:2 - 2 = 1:2 | 3 | 75.0 | 21.7 | 32.6 - 100.0 |
|  | 3:4 - 2 = 1:4 | 2 | 50.0 | 25.0 | 1.0 - 99.0 |
|  | 4:3 - 2 = 2:3 | 3 | 75.0 | 21.7 | 32.6 - 100.0 |
|  | 4:5 - 2 = 2:5 | 2 | 50.0 | 25.0 | 1.0 - 99.0 |
|  | 4:5 - 2 = 4:3 | 0 | 0.0 | 0.0 | 0.0 |
|  | 2:3 - 3 = 2:0 | 1 | 25.0 | 21.7 | 0.0 - 67.4 |
|  | 3:4 - 3 = 0:4 | 3 | 75.0 | 21.7 | 32.6 - 100.0 |
|  | 3:4 - 3 = 3:1 | 2 | 50.0 | 25.0 | 1.0 - 99.0 |
|  | 4:5 - 3 = 1:5 | 1 | 25.0 | 21.7 | 0.0 - 67.4 |
|  | 4:5 - 3 = 4:2 | 3 | 75.0 | 21.7 | 32.6 - 100.0 |
|  |  |  |  |  |  |
| **Yalinga** | 1:2 - 1 = 0:2 | 2 | 50.0 | 25.0 | 1.0 - 99.0 |
|  | 2:4 - 1 = 2:3 | 4 | 100.0 | 0.0 | 100.0 |
|  | 2:5 - 1 = 2:4 | 1 | 25.0 | 21.7 | 0.0 - 67.4 |
|  | 2:3 - 1 = 1:3 | 2 | 50.0 | 25.0 | 1.0 - 99.0 |
|  | 3:4 - 1 = 2:4 | 1 | 25.0 | 21.7 | 0.0 - 67.4 |
|  | 4:5 - 1 = 3:5 | 2 | 50.0 | 25.0 | 1.0 - 99.0 |
|  | 1:2 - 2 = 1:0 | 2 | 50.0 | 25.0 | 1.0 - 99.0 |
|  | 2:5 - 2 = 2:3 | 2 | 50.0 | 25.0 | 1.0 - 99.0 |
|  | 3:2 - 2 = 1:2 | 2 | 50.0 | 25.0 | 1.0 - 99.0 |
|  | 3:4 - 2 = 1:4 | 2 | 50.0 | 25.0 | 1.0 - 99.0 |
|  | 4:3 - 2 = 2:3 | 2 | 50.0 | 25.0 | 1.0 - 99.0 |
|  | 4:5 - 2 = 2:5 | 1 | 25.0 | 21.7 | 0.0 - 67.4 |
|  | 4:5 - 2 = 4:3 | 2 | 50.0 | 25.0 | 1.0 - 99.0 |
|  | 2:3 - 3 = 2:0 | 2 | 50.0 | 25.0 | 1.0 - 99.0 |
|  | 3:4 - 3 = 0:4 | 4 | 100.0 | 0.0 | 100.0 |
|  | 3:4 - 3 = 3:1 | 3 | 75.0 | 21.7 | 32.6 - 100.0 |
|  | 4:5 - 3 = 1:5 | 3 | 75.0 | 21.7 | 32.6 - 100.0 |
|  | 4:5 - 3 = 4:2 | 2 | 50.0 | 25.0 | 1.0 - 99.0 |

**Table S5. Individual performance as a function of Condition in Subsequent Event (SUBE) task.** Includes individual number of correct responses (Correct / total of 4), probability of success (Probability), the standard error (SE) and the confidence interval (CI).

| **Subject** | **Condition** | **Correct** | **Probability** | **SE** | **2.5 - 97.5% CI** |
| --- | --- | --- | --- | --- | --- |
| **Nakuru** | 1:4 ± 1 = 2:3 | 2 | 50.0 | 25.0 | 1.0 - 99.0 |
|  | 2:3 ± 1 = 3:2 | 3 | 75.0 | 21.7 | 32.6 - 100.0 |
|  | 0:5 ± 2 = 2:3 | 3 | 75.0 | 21.7 | 32.6 - 100.0 |
|  | 1:4 ± 2 = 3:2 | 3 | 75.0 | 21.7 | 32.6 - 100.0 |
|  | 0:5 ± 3 = 3:2 | 3 | 75.0 | 21.7 | 32.6 - 100.0 |
|  | 1:4 ± 3 = 4:1 | 3 | 75.0 | 21.7 | 32.6 - 100.0 |
|  |  |  |  |  |  |
| **Njano** | 1:4 ± 1 = 2:3 | 2 | 50.0 | 25.0 | 1.0 - 99.0 |
|  | 2:3 ± 1 = 3:2 | 3 | 75.0 | 21.7 | 32.6 - 100.0 |
|  | 0:5 ± 2 = 2:3 | 4 | 100.0 | 0.0 | 100.0 |
|  | 1:4 ± 2 = 3:2 | 2 | 50.0 | 25.0 | 1.0 - 99.0 |
|  | 0:5 ± 3 = 3:2 | 3 | 75.0 | 21.7 | 32.6 - 100.0 |
|  | 1:4 ± 3 = 4:1 | 2 | 50.0 | 25.0 | 1.0 - 99.0 |
|  |  |  |  |  |  |
| **Nuru** | 1:4 ± 1 = 2:3 | 1 | 25.0 | 21.7 | 0.0 |
|  | 2:3 ± 1 = 3:2 | 0 | 0.0 | 0.0 | 0.0 |
|  | 0:5 ± 2 = 2:3 | 4 | 100.0 | 0.0 | 100.0 |
|  | 1:4 ± 2 = 3:2 | 4 | 100.0 | 0.0 | 100.0 |
|  | 0:5 ± 3 = 3:2 | 4 | 100.0 | 0.0 | 100.0 |
|  | 1:4 ± 3 = 4:1 | 3 | 75.0 | 21.7 | 32.6 - 100.0 |
|  |  |  |  |  |  |
| **Yalinga** | 1:4 ± 1 = 2:3 | 2 | 50.0 | 25.0 | 1.0 - 99.0 |
|  | 2:3 ± 1 = 3:2 | 2 | 50.0 | 25.0 | 1.0 - 99.0 |
|  | 0:5 ± 2 = 2:3 | 2 | 50.0 | 25.0 | 1.0 - 99.0 |
|  | 1:4 ± 2 = 3:2 | 2 | 50.0 | 25.0 | 1.0 - 99.0 |
|  | 0:5 ± 3 = 3:2 | 2 | 50.0 | 25.0 | 1.0 - 99.0 |
|  | 1:4 ± 3 = 4:1 | 4 | 100.0 | 0.0 | 100.0 |
